# Malpractice

**Published:** 1845-01

**Authors:** 


*Malpractice.*—The London Lancet, of the 24th of August, contains an account of the following most extraordinary instance of malpractice, resulting in the death of its subject:

The name of the unfortunate patient was Mary Dent, wife of John Dent, of Littleport, in the Isle of Ely, laborer; she was twenty-three years of age, of good health, and robust appearance; she had borne five children, and had miscarried once; she was married at a very early age, and became a mother when between sixteen and seventeen years old.

On the 22d day of May last, Mary Dent complained of feeling very unwell; she felt great pain, and vomited occasionally, and being apprehensive of miscarriage, sent off, about 11 o'clock at night, for Mr. John Garland, a person of middle age, who has practised as a surgeon and accoucheur, at Littleport, ever since 1816.

It appears that on the day in question the patient had occasion to lift or drag a sack of flour, containing fourteen stone; it also appears that, according to her own account, she was at this time about three months advanced in pregnancy, having menstruated for the last time on the 14th of February.

Upon his arrival, Mr. Garland proceeded to examine the patient, by passing his hand and arm into the vagina, intending, as he himself expressed it, to "bring the child." He shortly afterwards made a second examination, whereupon the patient entreated of him to desist, "for he was pulling her entrails out;" and presently Ann Banyard, the nurse, saw hanging out in the bed "a large quantity of entrails, as many as could lie on a large plate." (I quote her own words as taken down by the coroner.)

When matters had arrived at this crisis, Mr. Garland appeared most anxious for further advice and assistance, and at his urgent request, a messenger was despatched to Ely, who returned, bringing with him Mr. Jones, of Ely, surgeon.

Upon turning down the bed-clothes, Mr. Jones discovered in the bed a something, which he at first mistook for the umbilical cord, but a more careful examination convinced him that the protruding mass was small intestine, depending from the vagina. Upon a minute inspection, he ascertained that the intestine was completely detached from the mesentery, throughout its whole length, and that it was extensively lacerated; the distal portion being torn completely across, that is, its whole diameter being completely divided, whilst the proximal portion was lacerated, so as to be very nearly divided. Under these unfortunate and perplexing circumstances, Mr. Jones was of opinion that any attempt to save the intestine would prove useless, he therefore passed a ligature around the intestine, above the proximal laceration, and close to the vagina, and cut off all the intestine below the ligature; the intestine, so cut off, measured nineteen feet six inches. Mr. Jones then took his departure, stating his belief that the patient could not survive many hours.

All this occurred between 4 and 7 o'clock, on the morning of the 23d of May. Shortly after the departure of Messrs. Jones and Garland, the fœtus was found in the bed; it appeared a fœtus of about three months. About twelve hours after the application of the ligature, the bowel above the ligature became very much distended, and ultimately burst. Subsequently the nurse, Ann Banyard, removed about half a yard more of intestine, without any medical man being present; she cut it off, and she did so "because it became very black and was very offensive."

The poor woman lingered seventeen days, and expired suddenly and at once, whilst attempting to raise herself up in bed, on the 8th day of June. During this period she did not suffer so acutely as might have been imagined; for three or four days the stomach rejected every thing, but latterly it became much less irritable; her skin was cool, her pulse rarely above eighty, her countenance natural, and she complained of no pain, neither was there any appearance of hæmorrhage. She took a little simple sesqui-carbonate of soda as medicine, and weak gruel or chicken-broth as diet.

I was instructed to make a *post mortem* examination, about forty-eight hours after death. The following is the record of the examination:

A very marked flatness and depression was observable between the two ilia; over the whole abdomen the bodies of the vertebræ could be more distinctly felt than naturally they should be; the external parts of generation and the perinæum were very much excoriated. There was nothing else worthy of note about the trunk.

Upon opening the abdomen, the liver was ascertained to be healthy, as also the stomach. The omentum was offensive, black, gangrenous, and adherent to the arch of the colon and to the small intestines generally; this adhesion was more marked from the symphysis-pubis to the right than to the left iliac region.

The colon appeared shrunken and contracted, and was so adherent to the omentum throughout the extent of the ascending and transverse portions, that the omentum and colon might be turned back together; the whole of the ascending portion of the colon was in a state of complete gangrene; over the region of the cæcum a small and circumscribed collection of matter was found, and it appeared as if, in this situation, the small intestines had been separated from the large; the descending portion of the colon and the rectum were not in so diseased a condition.

About *two yards* only of small intestines remained in the abdomen, these, towards the lower portion, were very gangrenous, and upon tracing them downwards it was discovered that they were very adherent in the right iliac region, and that in this situation they dipped downwards and inwards to the right of the uterus, and became attached, by their lower margin, to the borders of an opening found in the right side of the vagina; the small intestines appeared to terminate in the vagina, for they could not be traced onwards to the large.

The mesentery was gangrenous, and had been torn in two or three places.

In the vagina was found, in the upper part and on the right side, a laceration sufficiently large to admit two or three of my fingers; this laceration was found to communicate with, and to lead into, the above-mentioned depending portion of small intestine, so that the fingers could readily be passed from the vagina into the small intestine; the vagina on the left side was healthy and unruptured.

The uterus was normal in size and appearance, and what perhaps is rather singular, did not exhibit any traces of recent impregnation.

The bladder was healthy, so were the lungs, and so was the heart.

The jury found Mr. Garland *guilty*, and he was sentenced to one month's imprisonment.

